# The Role of Long Noncoding RNA AL161431.1 in the Development and Progression of Pancreatic Cancer

**DOI:** 10.3389/fonc.2021.666313

**Published:** 2021-07-30

**Authors:** Gang Ma, Guichen Li, Wufeng Fan, Yuanhong Xu, Shaowei Song, Kejian Guo, Zhe Liu

**Affiliations:** Department of Pancreatic-Biliary Surgery, First Hospital of China Medical University, Shenyang, China

**Keywords:** long noncoding RNA, pancreatic cancer, epithelial mesenchymal transition (EMT), bioinformatics, diagnosis

## Abstract

Pancreatic cancer is known for its notorious fast progression and poor prognosis. Long noncoding RNA (lncRNA) AL161431.1 has been reported to be involved in the pathogenesis of different cancers. In this study, we explored the role of lncRNA AL161431.1 in the development and progression of pancreatic cancer by bioinformatic analysis, *in vitro* and *in vivo* experiments in pancreatic cancer BxPC-3 and SW1990 cells, as well as clinical samples. We found that lncRNA AL161431.1 was highly expressed in pancreatic cancer cells and tissues. Knock down of lncRNA AL161431.1 led to increased cancer cell death and cell cycle arrest. Xenograft growth of SW1990 cells with stable knockdown of lncRNA AL161431.1 in mice was significantly slower than that of SW1990 cells with scrambled control shRNA. Finally, we showed the involvement of lncRNA AL161431.1 in pancreatic cancer was related to its promotion of epithelial mesenchymal transition process.

## Introduction

In the United States, pancreatic cancer represents about 3% of cancers and 7% of cancer-related deaths (1). About 57,600 people were predicted to be diagnosed with pancreatic cancer, with 47,050 deaths according to the estimate for pancreatic cancer for 2020 from the American Cancer Society in the United States. The 5-year relative survival rate of patients with pancreatic cancers at all stages was only 9% based on the survey on patients diagnosed with pancreatic cancer between 2009 and 2015 (2). Furthermore, pancreatic cancer often presents at late stage, and only 20% of patients showed surgically resectable lesions at diagnosis (3). Therefore, better understanding the mechanisms of tumorigenesis and development of pancreatic cancer is pivotal to its early diagnosis and improved prognosis.

Long noncoding RNAs (lncRNAs) are mRNA transcripts longer than 200 nucleotides that are not destined to be translated into proteins (4). Although thought to be non-functional for decades, more and more evidence has shown that lncRNAs are critical regulators of pathogenesis, progression, and metastasis of a broad scope of cancers (5). Many lncRNAs are promising diagnostic and prognostic biomarkers for cancers, including pancreatic cancer (6), whereas lncRNA AL161431.1 has been reported to be involved in the pathogenesis of endometrial carcinoma (7), hypoxic TCGA-BRCA tumors (8), and lung squamous cell carcinoma (9).

In the present study, we explored the role of lncRNA AL161431.1 in the development and progression of pancreatic cancer by employing bioinformatic analysis and *in vitro* and *in vivo* experiments in two different pancreatic cancer cell lines as well as clinical samples. We showed that lncRNA AL161431.1 was highly expressed in pancreatic cancer cell lines and clinical tissues. Knocking down of lncRNA AL161431.1 led to increased cancer cell death and cell cycle arrest. The involvement of lncRNA AL161431.1 in pancreatic cancer progression was related to its promotion of the epithelial mesenchymal transition (EMT) process.

## Materials and Methods

### Bioinformatic Analysis

The transcriptome sequencing data of 181 samples were generated by the R package in The Cancer Genome Atlas- Pancreatic adenocarcinoma (TCGA-PAAD) dataset obtained from the TCGAbiolinks (https://bioconductor.org/packages/release/data/experiment/vignettes/TCGAbiolinksGUI.data/inst/doc/vignettes.html). Differential mRNA abundance analyses were carried out using DESeq2 (http://www.r-project.org/). Genes with reads < 5 in any sample were filtered out from the final quantitative analysis. Heatmap and volcano plot were constructed by normalized gene expression *via* R package. The normalized gene expressions were subjected to Gene Set Variation Analysis (GSVA, a non-parametric, unsupervised method for estimating variation of gene set enrichment through the samples of an expression dataset). R package survival was used for overall survival analysis. Cox proportional hazard (PH) model was executed by the functions of survival and survminer in R package. The best-scanned cutoff points are defined as the one with the most significant (log-rank test) split. R package survival ROC was used for Receiver Operating Characteristic (ROC) curve and Area Under Curve (AUC) plotted for different durations of survival analysis.

### Patients

Ethical approval was obtained from the Ethical Committee of the First Affiliated Hospital of China Medical University (a tertiary hospital and regional cancer center in Shenyang, China). Written informed consent was obtained from all patients at admission. The diagnosis of pancreatic cancer was based on the National Comprehensive Cancer Network Guidelines (NCCN 2018), and no patient received preoperative chemotherapy. Pancreatic cancer tissues and adjacent normal tissue from the same patients were obtained during pancreaticoduodenectomy at the First Affiliated Hospital of China Medical University from June 1, 2018 to May 31, 2019, who had no bacterial or viral infections, chronic diseases such as hypertension, cardiovascular diseases, diabetes, or other cancers. Tissue samples were cryopreserved in liquid nitrogen immediately after surgical resection until further experiments.

### Cell Culture

All experiments were performed with mycoplasma-free cells. Human pancreatic adenocarcinoma cell lines PANC-1 (RRID : CVCL_0480), BxPC-3 (RRID : CVCL_0186), and SW1990 (RRID : CVCL_1723) were purchased from Shanghai Zhong Qiao Xin Zhou Biothechnology Co., Ltd (Shanghai, China). Cells were cultured in Dulbecco’s modified Eagle’s medium (DMEM, Gibco, Thermo Fisher Scientific, Inc.) supplemented with 10% fetal bovine serum (FBS, Gibco), 10 mM HEPES (Gibco), 2 mM L-glutamine (Gibco), 1 mM pyruvate sodium (Gibco), 100 U/ml penicillin and 100 µg/ml streptomycin (Gibco) at 37°C with 5% CO_2_.

### Stable lncRNA-Knockdown Cell Line Construction

A specific SW1990 cell line (SW1990-LNC-KD) with stable knockdown of lncRNA AL161431.1 or control SW1990 cell line was constructed using a lnc-shRNA sequence targeting 5’- GCAGTATTCCTGCACTTCT -3’ or scramble control sequence 5’- TTCTCCGAACGTGTCACGT -3’ cloned into the pLV3(H1/GFP&Puro) vector (**Figure S1**), and transfected into 293T cells (Shanghai GenePharma China). After 72 h incubation, cell supernatants were collected, and used for transfection (24 h) of SW1990s (10). SW1990 cells containing the lnc-shRNA were selected by media containing 5 μg/ml puromycin (Sigma, St. Louis, MO), and were used for Xenograft tumor growth experiments (11).

### Transfection of siRNAs

Small interfering RNAs (siRNAs) and scrambled negative control for lncRNA were provided by Shanghai GenePharma (Shanghai, China). The siRNAs (30 nM of each, sequences listed in **Table S1**) were transfected with X-tremeGENE siRNA transfection reagent (Roche Applied Science, Shanghai, China) according to the manufacturer’s manual.

### Total RNA Extraction and qRT-PCR

Fresh cells or frozen tissues were homogenized in TRIzol reagent (Thermo Fisher Scientific, Inc.) for total RNA extraction following the manual’s instructions. The purified RNAs were quantified by a NanoDrop 2000 Spectrophotometers (Thermo Fisher Scientific, Inc.), reverse transcribed using an RT reagent Kit (Nachuan Bio-Tech Co., Binzhou, China) based on the manufacturer’s instructions. qRT-PCR analysis was performed in an Exicycle 96 Real-Time Quantitative Thermal Block (Bioneer) using SYBR Green Master Mix (Nachuan Bio-Tech Co.) according to the manufacturer’s protocol (sequence of primers listed in **Table S1**). Reactions were performed in 96-well plates in 10 µL total volume including 1x SYBR Green Master mix, cDNA (10 ng), and primers (75 nM of forward and reverse primers each). The average of triplicate qRT-PCR results of target lncRNA expression from each sample was normalized by β-actin of the same sample, and the relative expression was calculated using 2^−ΔΔCt^.

### Cell Count Kit-8 Assay for Cell Proliferation

Cell proliferation was detected using a CCK-8 assay kit (DOJINDO, Japan). Cells were seeded into 96-well plates (1 x 10^4^/well). At 10 am, 10 μl CCK-8 reagent in 100 μl medium was added to each well. The light absorbance at 450 nm of each well was measured after 2 hour incubation at 37°C with an Epoch microplate spectrophotometer (BioTek China, Beijing). Cell viability was monitored in triplicate experiments and expressed as a percentage of that of the control cells.

### Flow Cytometry

Forty-eight hours after siRNA transfection, cell death/apoptosis or cell cycle was analyzed by flow cytometry (LSR, BD Biosciences) using Annexin V-FITC/PI Apoptosis Detection Kit (Beyotime Institute of Biotechnology, Shanghai, China) or Propidium Iodide (PI; Solarbio Biotech, China), respectively, according to the manufacturer’s instructions. For cell death/apoptosis, cells were collected, washed three times with cold PBS, stained in 500 μl staining buffer (Annexin V-FITC/PI in PBS) at room temperature for 30 min in the dark. For cell cycle analysis, cells were collected, washed three times with cold PBS, fixed in precooled anhydrous ethanol at 4°C for 30 min, and stained with 50 ug/mL PI in 500 μl PBS in the dark. All experiments were triplicated.

### Immunofluorescence Staining

Cells were grown on sterile glass slides, fixed with 4% paraformaldehyde for 30 min, blocked with 1% BSA for 30 min, incubated with primary antibodies against CDH1 (E-cadherin, AF0131, 1:500; Affinity Biosciences LTD.), CDH2 (N-cadherin AF4039, 1:200; Affinity Biosciences LTD.), and VIM (Vimentin AF7013, 1:250; Affinity Biosciences LTD.) at 4°C overnight, followed by incubation with Alexa Fluor^®^ 594 conjugated Affinipure Goat Anti-Rabbit IgG (H + L) secondary antibodies (Jackson ImmunoResearch Laboratories, PA) for 1 hour. Nuclei were counterstained with DAPI. Images were captured with Olympus IX81 inverted fluorescence microscope (Olympus, Beijing, China).

### Western Blotting

Anti-beta actin (AF7018, 1:3000), anti-E-cadherin (AF0131, 1:10000), anti-Vimentin (AF7013, 1:1000), anti-N-cadherin (AF4039, 1:1000) were obtained from Affinity Biosciences LTD., and the secondary antibody concentration was 1:5000 (S0001, Affinity Biosciences LTD.). Forty-eight hours after siRNA transfection, cells were lysed in RIPA lysis buffer (Merck Group, Germany) for 30 min on ice. Sample protein concentrations were quantified using a BCA assay kit (Solarbio, China). Equal amounts of proteins were separated by SDS-polyacrylamide gel electrophoresis, transferred to polyvinylidene difluoride membranes, and probed with the antibodies of interest. The intensity of bands was quantified using ImageJ, using β-actin as internal loading control.

### Xenograft Tumor Growth in Nude Mice

Animal experiment protocols were approved by the Institutional Ethical Committee, and animals were properly treated in accordance with the institutional ethical requirements of experimental animals. Male nude mice were kept in a temperature-controlled specific-pathogen-free animal laboratory, with a 12h light/dark cycle. All animals had free access to food and water. SW1990-LNC-KD cells (1.5 × 10^6^ cells in 0.1 ml sterile PBS, with stable knockdown of lncRNA AL161431.1), or SW1990-LNC-NC cells (with scrambled shRNA) were subcutaneously injected into the left flank of mice at 8 weeks of age. The volume of tumor was measured every morning by length x width x depth in mm. The mice were euthanized 2 weeks after cancer cell injection, and the growth of subcutaneous tumors were compared (n=7 in each group).

### Immunohistochemistry

PCNA immunohistochemical staining was performed on 10 μm sections of paraffin-embedded tissue samples. In brief, the slides were incubated in PCNA antibody (AF0239, Affinity Biosciences LTD.; at 1:100 dilution) at 4°C overnight followed by the secondary antibody (S0001, Affinity Biosciences LTD.; at 1:200 dilution) at 37°C for 1 hours. Nuclei were counterstained with hematoxylin. PCNA positive cells were quantified using the IHC Profiler (12).

### Transwell Assay

Twenty-four hours after transfection, SW1990 (1x10^5^) or BxPC-3 (1x10^5^) cells were resuspended in 100 µl serum-free medium and seeded into the upper chamber with 12 µm pore polycarbonate membranes pre-coated with Matrigel Basement Membrane Matrix (BD Biosciences, Bedford, MA). The lower chamber was filled with 600 μl of 1640 medium supplemented with 20% FBS. After 24 hours of incubation at 37°C with 5% CO_2_, cells remaining in the upper membrane surface were removed with a cotton swab, whereas invaded cells were fixed and stained with 0.5% crystal violet (13).

### Wound-Healing Assay

Twenty-four hours after transfection, cells were seeded in 24-well plates at 1x10^5^ cells/well. Cells were cultured in medium containing 5% FBS with 5% CO_2_ for 24 h. A 1-mm wide scratch was made in the confluent cultures with a pipette tip, followed by wash twice with PBS to remove debris. The area of the scratch was measured using images taken by a phase-contrast microscope (14).

### Statistics

Statistical analysis was performed using SPSS software (version 24, Armonk, NY: IBM Corp.). Quantitative data are presented as mean ± SD. Differences in the mean of two samples were analyzed by Student’s t-test. All 2-tailed statistical tests were considered significant when p < 0.05.

## Results

### LncRNA AL161431.1 Was Overexpressed in Pancreatic Adenocarcinoma

**Figure 1:**
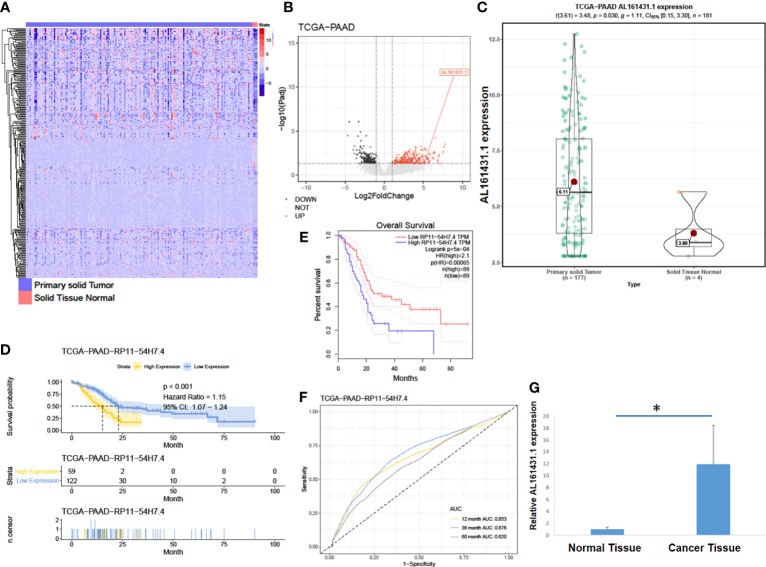
LncRNA AL161431.1 was overexpressed in pancreatic adenocarcinoma. **(A)** Heatmap of TCGA-PAAD transcriptome; **(B)** Volcano plot of different gene expressions in TCGA-PAAD; **(C)** LncRNA AL161431.1 expression in the TCGA-PAAD cohort; **(D, E)** Kaplan-Meier curves of the overall survival in the TCGA-PAAD cohort; **(F)** ROC curves of patients in the TCGA-PAAD cohort; **(G)** Relative AL161431.1 expression in pancreatic cancer and normal tissues from clinical samples (qRT-PCR). *p < 0.05.

In order to identify lncRNAs that are most relevant to pancreatic cancer, we obtained data on tumor (PAAD) transcriptome-level 3 (including 177 pancreatic cancer tissues and 4 normal pancreatic tissues) from the TCGA database (15). The expression of lncRNA AL161431.1 (also named RP11-54H7.4) in pancreatic cancer tissues was found to be significantly higher than that of normal pancreatic tissues (**Figures 1A–C**) by DESeq2 software package (16) analysis. We further analyzed the data of TCGA-PAAD cohort by dividing the samples into a high- and a low- lncRNA AL161431.1 expression groups using the median as the cut-off value. Using GEPIA online tool (17) and R language survival analysis, it was found that patients in the high lncRNA AL161431.1 expression group had a shorter survival period (**Figures 1D, E**), and lncRNA AL161431.1 was shown to be positively correlated with the prognosis of pancreatic adenocarcinoma (**Figure 1F**). Pancreatic cancer tissues (n=26) and paired adjacent normal tissue from the same patients (n=26) were obtained from 14 male and 12 female patients admitted in our hospital aged between 43 and 63 (mean age 53.8 ± 6.0 years) and analyzed by qRT-PCR. According to the eighth edition of the AJCC-TNM staging system for pancreatic cancer, there were 6 cases in the first stage, 12 cases in the second stage, 7 cases in the third stage, and 1 case in the fourth stage. Among the 26 cases, 10 were poorly differentiated, 8 were moderately differentiated, and 8 were highly differentiated. The level of lncRNA AL161431.1 was significantly higher in cancer tissues than that of the normal tissues (p<0.05, **Figure 1G**). The above results suggested that lncRNA AL161431. 1 was highly expressed in pancreatic cancer tissues, and overexpression of lncRNA AL161431. 1 could indicate a poor prognosis.

### Knocking Down of lncRNA AL161431.1 Inhibited the Growth, Invasion and Migration of Pancreatic Adenocarcinoma Cells

**Figure 2:**
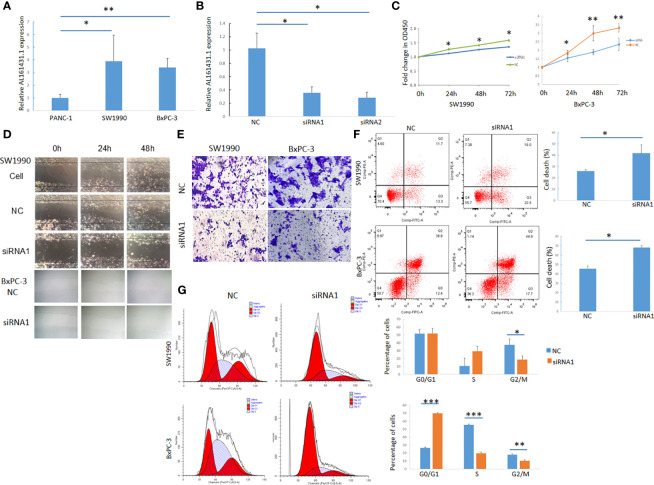
Knockdown of lncRNA AL161431.1 inhibited the growth, invasion and migration and promoted apoptosis in pancreatic adenocarcinoma cells. **(A)** Relative AL161431.1 expression in PANC-1, SW1990, and BxPC-3 cells analyzed by qRT-PCR; **(B)** Relative AL161431.1 expression in SW1990 cells after siRNA transfection analyzed by qRT-PCR; **(C)** CCK-8 assay showing fold change at OD450, representing growth curve of SW1990 and BxPC-3 cells at 24, 48, and 72 hours after siRNA1 transfection; **(D)** Wound healing assay in naïve, scramble siRNA, or siRNA1 transfected SW1990 or BxPC-3 cells at 0, 24, and 48 hours post scratching (100x); **(E)** Transwell assay in scrambled control siRNA or siRNA1 transfected SW-1990 or BxPC-3 cells (100x); **(F)** Cell death (Q2+Q3) in scrambled control siRNA or siRNA1 transfected SW1990 and BxPC-3 cells analyzed by flow cytometry; **(G)** Cell cycle analysis of SW1990 and BxPC-3 cells after transfection of scrambled control siRNA or siRNA1 by flow cytometry. NC: scrambled control siRNA or shRNA. *p < 0.05, **p < 0.01, ***p < 0.001.

The expression of lncRNA AL161431.1 in pancreatic cancer cell lines PANC-1, SW1990 and BxPC-3 cells was determined by qRT-PCR, and was found to be significantly higher in SW1990 and BxPC-3 cells compared to that in PANC-1 cells (**Figure 2A**). In order to study the function of AL161431.1, we tested specific siRNAs to knock down the expression of lncRNA AL161431.1, and found both siRNA1 and siRNA2 had significant knockdown effect (p<0.05 for both, **Figure 2B**). siRNA1 was selected for the following knockdown experiments. CCK-8 assay showed that the cell proliferative activity in the AL161431.1 siRNA knockdown group was significantly lower than that in the control group in both SW1990 and BxPC-3 cell lines (**Figure 2C**). Wound healing and Transwell experiments showed that after knocking down lncRNA AL161431.1, cell migration and invasion was significantly inhibited (**Figures 2D, E**). Compared with the scrambled control group, siRNA knock down of AL161431.1 significantly promoted cell death (**Figure 2F**), and the cells in the knockdown group accumulated in G1/S phase (**Figure 2G**). These results suggested that knocking down LncRNA AL161431.1 can inhibit the growth, invasion and migration, and promote apoptosis in pancreatic adenocarcinoma cells.

### LncRNA AL161431.1 in Nude Mice Xenografts

**Figure 3:**
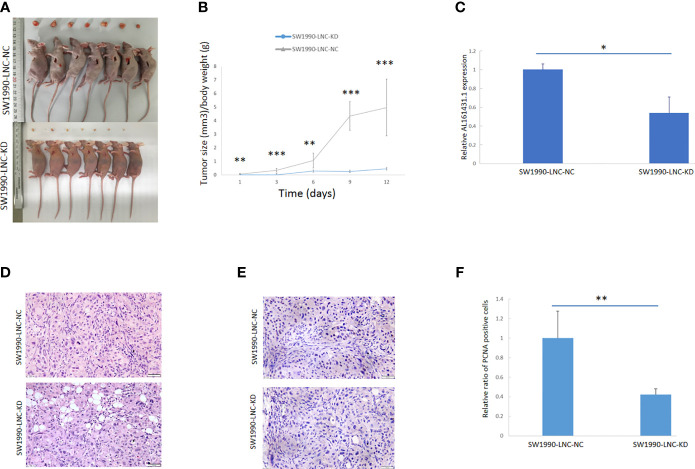
Knockdown of lncRNA AL161431.1 inhibited the growth of tumor xenografts. **(A)** Size of SW1990 tumor xenografts with SW1990-LNC-KD or SW1990-LNC-NC cells at 14 days after injection; **(B)** Time course of in *vivo* xenografts growth measured in mm^3^; **(C)** Relative AL161431.1 expression in engrafted tumors with SW1990-LNC-NC cell or SW1990-LNC-KD cell; **(D)** HE staining of tumor xenografts; **(E)** PCNA immunohistochemistry in tumor xenografts; **(F)** Relative ratio of PCNA positive cells in the engrafted tumors with SW1990-LNC-NC cell or SW1990-LNC-KD cell. *p < 0.05, **p < 0.01, ***p < 0.001.

The above experiments have demonstrated that knockdown of lncRNA AL161431.1 can inhibit cell growth *in vitro*. In order to further evaluate the role of AL161431.1 *in vivo*, we constructed the lncRNA AL161431.1 stable knockdown cell line (SW1990-LNC-KD cells) using specific shRNA. The cells in the 8th passage was found to have minimum residual level of lncRNA AL161431.1 compared with the 3rd and 5th passages by qRT-PCR analysis (data not shown), and was injected into nude mice for xenograft tumor growth analysis. The size of xenograft tumor in the stable knockdown group was significantly smaller than that of the control group at 2 weeks post injection (**Figure 3A**). The knockdown group also showed slower tumor growth (**Figure 3B**). The expression of lncRNA AL161431.1 in the tumor tissue was lower in the knockdown group (**Figure 3C**). HE staining and PCNA (cell proliferation index) immunohistochemical staining showed reduced cell atypia and fewer PCNA-positive cells in the knockdown group (**Figures 3D, E**). There was significant lower number of PCNA-positive cells in SW1990-LNC-KD cell xenograft tumor tissue (p<0.01; **Figure 3F**). These results suggested that knocking down of lncRNA AL161431.1 inhibited the growth of xenograft tumors.

### Predicting the Function of lncRNA AL161431.1 in Pancreatic Cancer Cells

**Figure 4:**
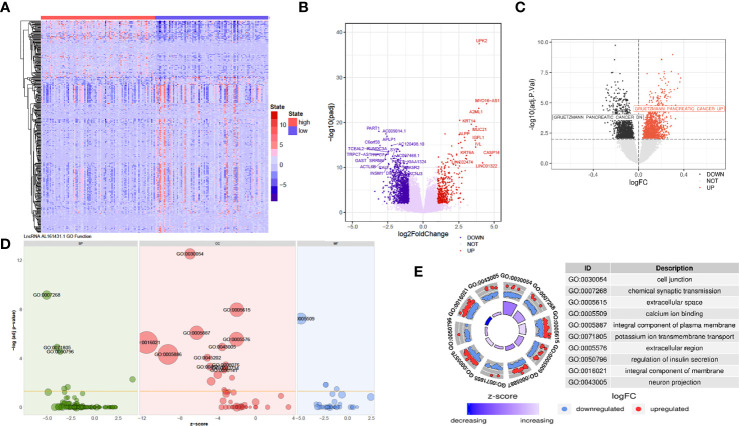
Prediction of the function of lncRNA AL161431.1 in cells. Heatmap **(A)** and Volcano plot **(B)** of different gene expression between high- and low- AL161431.1 expression patients in the TCGA-PAAD cohort; **(C)** GSVA analysis of lncRNA AL161431.1 effect on pancreatic adenocarcinomas in TCGA cohort; **(D, E)** GO function analysis of AL161431.1.

The above *in vivo* and *in vitro* studies suggested that lncRNA AL161431.1 promoted cell growth. Using the median of lncRNA AL161431.1 as the cut-off value threshold, the expression of differential genes was analyzed by the DEseq2 (**Figures 4A, B**), and GSVA (**Figure 4C**). The enrichment of differentially expressed genes and the known gene set was analyzed by the GSEA software. It was found that genes that were upregulated in pancreatic cancer were highly enriched in the AL161431.1 high expression group. Through GO enrichment analysis (18), AL161431.1 was found to play roles in various cellular functions and processes such as cell-cell junction and extracellular microenvironment (**Figures 4D, E**).

### The Mechanism of lncRNA AL161431.1 in Regulating Cell Function

**Figure 5:**
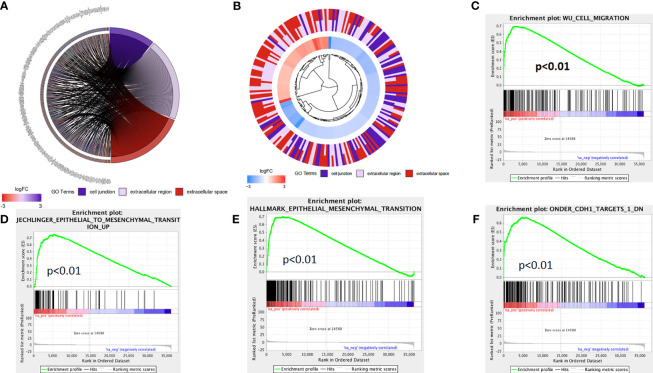
The mechanism of lncRNA AL161431.1 in regulating cell function. GO function relative genes **(A)** and fold changes **(B)**; **(C–F)** GSEA analysis for different gene expression in patients with high- and low- AL161431.1 expression in TCGA-PAAD.

The above analysis indicated that lncRNA AL161431.1 regulated cell migration and infiltration. We further analyzed the role of lncRNA AL161431.1 in regulating cell-cell junctions, and extracellular region. We found that there was an interaction network centered by lncRNA AL161431.1 (**Figures 5A, B**). Moreover, through GSEA analysis, we found that the differential genes of the high lncRNA AL161431.1 expression groups were highly enriched in the WU_CELL_MIGRATION gene set (**Figure 5C**), which was closely associated with the EMT (**Figures 5D–F**) and involved in cell migration and invasion. These bioinformatic analysis suggested that lncRNA AL161431.1 could potentially regulate the migration and invasion of pancreatic cancer cells through EMT process.

**Figure 6:**
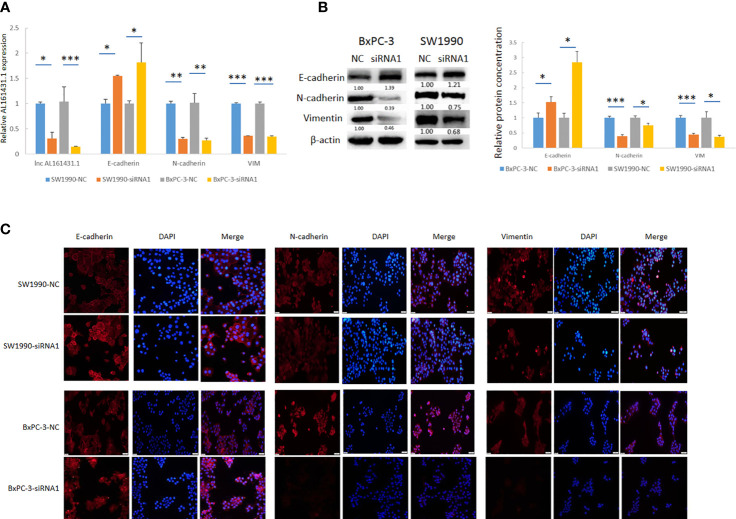
Knockdown of lncRNA AL161431.1 inhibited EMT. **(A)** qRT-PCR showing the knock down of lncRNA AL161431.1, and the corresponding changes in E-cadherin, N-cadherin, and vimentin in SW1990 and BxPC-3 cells; **(B)** Western blots showing the changes in protein level in E-cadherin, N-cadherin, and vimentin in SW1990 and BxPC-3 cells after transfection of scrambled siRNA or siRNA1; **(C)** Immunofluorescence analysis showing the change of expression of E-cadherin, N-cadherin, and vimentin in SW1990 and BxPC-3 cells after transfection of scrambled siRNA or siRNA1 (200x). *p < 0.05, **p < 0.01, ***p < 0.001.

In order to further verify the above bioinformatic findings on the role of lncRNA AL161431.1 in cell migration and invasion, we knocked down the expression of lncRNA AL161431.1 with siRNA1 in both SW1990 and BxPC-3 cells and the gene knockdown efficiency was confirmed by qRT-PCR (**Figure 6A**). Using qRT-PCR (**Figure 6A**), Western blot (**Figure 6B**) and immunofluorescence (**Figure 6C**) analyses, we found significant increase in the expression of CDH1 (E-cadherin), and decrease in the expression of CDH2 (N-cadherin) and VIM (Vimentin) in both mRNA and protein level after knocking down of lncRNA AL161431.1. These results suggested that lncRNA AL161431. 1 promoted cell migration and invasion by promoting EMT process.

## Discussion

LncRNAs have been shown to be involved in the development and progression of pancreatic cancer. A search using the key words of lncRNA and pancreatic cancer in PubMed on July 28, 2020 yielded 589 publications, with the most relevant one dating back in 2011 (19). Most lncRNAs have been reported to promote the proliferation of pancreatic cancer through different signaling pathways (20–23). In the case of lncRNA AL161431.1, its identified targets include miR-1252-5p, and the pathways include MAPK signaling in endometrial carcinoma and a competing endogenous RNA network in lung squamous cell carcinoma (7, 9; **Table S2**).

The employment of bioinformatics analyses was beneficial in identifying both candidate lncRNAs and lncRNAs-associated signaling pathways for mechanistic and therapeutic studies. Based on the analysis of TCGA-PAAD dataset, we found an increase of lncRNA AL161431.1 in clinical pancreatic cancer tissues, as well as in pancreatic cancer SW1990 and BxPC-3 cells. To test if this phenomenon was an coincidence, siRNA or shRNA were used to knock down lncRNA AL161431.1 in those cells, with the finding of significantly more cell death and cell cycle arrest, indicating that lncRNA AL161431.1 is truly involved in pancreatic cancer growth and progression.

EMT is crucial in tumorigenesis by enhancing metastasis and cancer cell stemness. The signature of EMT is the upregulation of Vimentin and N-cadherin accompanied by the downregulation of E-cadherin. EMT is a process which is regulated by a complicated network of signaling pathways and transcription factors, including the TGF- β signaling pathway, the MAPK signaling pathway, the JAK/STAT signaling pathway, the Hedgehog signaling pathway, the Wnt signaling pathway, the Hippo-YAP/TAZ signaling pathway, etc. (24). E-cadherin plays an important role in the initiation and maintenance of EMT, where cleavage of E-cadherin leads to the destabilization of cell-cell junctions as well as the release of β-catenin as a transcriptional activator for cell proliferation (25). Increased level of vimentin and decreased level of E-cadherin was evident in lung cancer cells (26). Vimentin has been reported as the main intermediate filament protein of normal mesenchymal tissue (27) with a major role of sustaining cellular integrity (28). Furthermore, vimentin expression was a potential independent adverse prognostic molecular biomarker in patients with pancreatic ductal adenocarcinoma (29). Similar to our recent findings of lncRNA ELFN1-AS1 (30), the finding of increased E-cadherin accompanied by decreased N-cadherin and vimentin in lncRNA AL161431.1 knockdown pancreatic cancer cells clearly indicated that lncRNA AL161431.1 is critical in the EMT process.

In summary, we showed by bioinformatics analysis, *in vivo* and *in vitro* experiments that lncRNA AL161431.1 was involved in the pathogenesis of pancreatic cancer by promoting EMT. LncRNA AL161431.1 could be a potential prognostic predictor and treatment target in pancreatic cancer.

## Data Availability Statement 

The original contributions presented in the study are included in the article/**Supplementary Material**. Further inquiries can be directed to the corresponding author.

## Ethics Statement 

The studies involving human participants were reviewed and approved by Ethical Committee of the First Affiliated Hospital of China Medical University. The patients/participants provided their written informed consent to participate in this study. The animal study was reviewed and approved by Ethical Committee of the First Affiliated Hospital of China Medical University.

## Author Contributions 

GM and ZL designed the study, analyzed and interpreted patient data, and were major contributors in writing the manuscript. GL and WF performed the cell culture, qRT-PCR and Western blot experiments. YX and SS performed flow cytometry, IF, and IHC experiments. KG performed xenografts experiments. All authors contributed to the article and approved the submitted version.

## Funding

(I) Liaoning Provincial Department of Education science research project (L2014299); (II) National Natural Science Foundation of China (81572360).

## Conflict of Interest

The authors declare that the research was conducted in the absence of any commercial or financial relationships that could be construed as a potential conflict of interest.

## Publisher’s Note

All claims expressed in this article are solely those of the authors and do not necessarily represent those of their affiliated organizations, or those of the publisher, the editors and the reviewers. Any product that may be evaluated in this article, or claim that may be made by its manufacturer, is not guaranteed or endorsed by the publisher.

## Supplementary Material

Supplementary Table 1 Primers for qRT-PCR and siRNAs.

Supplementary Table 2 The roles of LNC RNA AL161431.1 in different steps/mechanisms of the development and progression of different cancers.
