# Supplementary material for: The Role of Long Noncoding RNA AL161431.1 in the Development and Progression of Pancreatic Cancer
Source: Front Oncol. 2021 Jul 30;11:666313. doi: 10.3389/fonc.2021.666313 (PMC8363261; doi:10.3389/fonc.2021.666313)
Supplement: Supplementary Table 1 — Primers for qRT-PCR and siRNAs. [file Table_1.docx]

Table S1. Primers for qRT-PCR and siRNAs.

| qRT-PCR |  |  |
| --- | --- | --- |
| AL161431.1 | Forward | CTTTGAGCAAGGTCCGCAAG |
| AL161431.1 | Reverse | AGGTACCACAGGAGGCACAA |
| Gene knock out |  |  |
| siRNA-1 | AL161431.1 | sense : GCAGUAUUCCUGCACUUCUTT  antisense: AGAAGUGCAGGAAUACUGCTT |
| siRNA-2 | AL161431.1 | sense : CCCUUUCCAAUGCCUAGAUTT  antisense: AUCUAGGCAUUGGAAAGGGTT |
| siRNA-NC | Non mammal | sense : UUCUCCGAACGUGUCACGUTT  antisense: ACGUGACACGUUCGGAGAATT |
