# Supplementary material for: The Role of Long Noncoding RNA AL161431.1 in the Development and Progression of Pancreatic Cancer
Source: Front Oncol. 2021 Jul 30;11:666313. doi: 10.3389/fonc.2021.666313 (PMC8363261; doi:10.3389/fonc.2021.666313)
Supplement: Supplementary Table 2 — The roles of LNC RNA AL161431.1 in different steps/mechanisms of the development and progression of different cancers. [file Table_2.docx]

**Table S2. The roles of LNC RNA AL161431.1 in different steps/mechanisms of the development and progression of different cancers.**

| Study | Cancer type | Targets | Pathway | Application |
| --- | --- | --- | --- | --- |
| Gu, 2020 | Endometrial carcinoma | miR-1252-5p | MAPK signaling | Therapeutic |
| Ju, 2020 | Lung squamous cell carcinoma | NA | Competing endogenous RNA network | Prognosis |
| This study | Pancreatic cancer | E-cadherin, N-cadherin, vimentin | EMT | Pathogenesis, prognosis, therapeutic |
