# Supplementary figures and images for: The Role of Long Noncoding RNA AL161431.1 in the Development and Progression of Pancreatic Cancer
Source: Front Oncol. 2021 Jul 30;11:666313. doi: 10.3389/fonc.2021.666313 (PMC8363261; doi:10.3389/fonc.2021.666313)

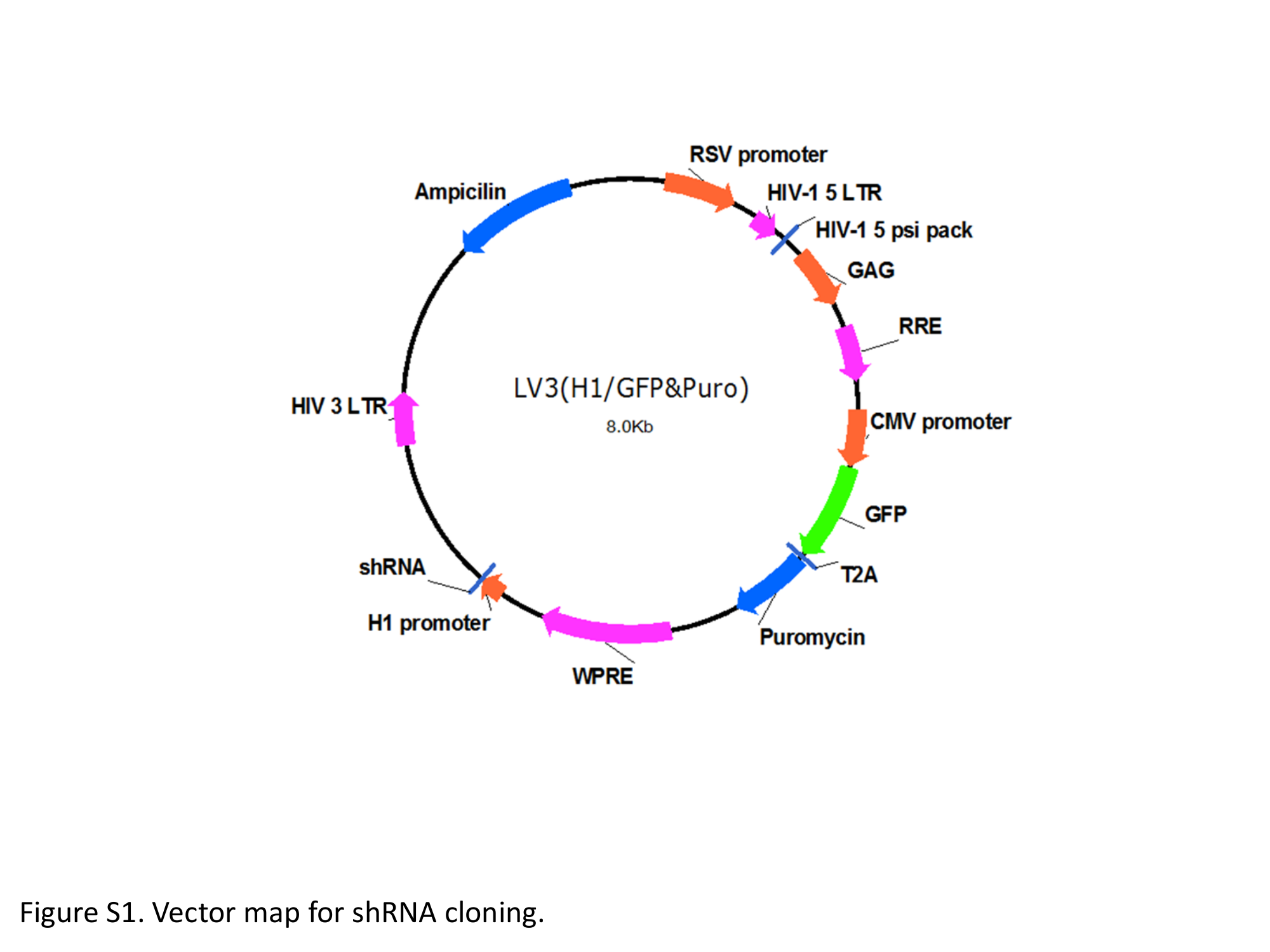

Supplement: Supplementary file 3 [file Image_1.tiff]
